# COMPARISON OF NURSING HOME STAFF’S PERCEPTIONS OF NOISE TO MEASURED NOISE LEVELS

**DOI:** 10.1093/geroni/igz038.1027

**Published:** 2019-11-08

**Authors:** April Ames, Stacie Dubin, Michael Valigosky, Victoria Steiner

**Affiliations:** University of Toledo, Toledo, Ohio, United States

## Abstract

A noisy environment may affect the ability of healthcare staff in nursing facilities to effectively complete tasks and provide quality care to residents. Staff may also become irritable or annoyed due to their perception that noise levels are too loud. The purpose of this descriptive study was to examine the differences in nursing home staff’s perceptions of noise levels compared to measured noise levels in four nursing home facilities in Ohio. A questionnaire was also distributed to examine the perceptions of noise levels by staff and the effects of noise on their health. The majority of the respondents (n=90) were white females. They described all facilities as being moderately noisy which was consistent with the measured noise levels. The loudest perceived noise sources included door/patients alarms and floor cleaners, which was confirmed by measured noise levels. The majority of facilities identified the nurses station as one of the noisiest locations; however, this was inconsistent with measured noise levels. Overall, respondents at all facilities felt neutral or disagreed that the noise levels impacted themselves or the residents. However, some respondents agreed that in a noisy environment it is easier to make job errors, difficult to concentrate on work, and they find themselves irritable or agitated. Perceptions of noise should be considered along with measured noise levels because tolerance levels differ among individuals and mental activities involving memory or complex analysis are sensitive to noise which may affect job performance.

